# *Bordetella petrii* Infection with Long-lasting Persistence in Human

**DOI:** 10.3201/eid1704.101480

**Published:** 2011-04

**Authors:** Alain Le Coustumier, Elisabeth Njamkepo, Vincent Cattoir, Sophie Guillot, Nicole Guiso

**Affiliations:** Author affiliations: Centre Hospitalier, Cahors, France (A. Le Coustumier);; Institut Pasteur, Paris, France (E. Njamkepo, S. Guillot, N. Guiso);; Centre Hospitalier Universitaire, Côte de Nacre, Caen, France (V. Cattoir)

**Keywords:** Bordetella petrii, bacteria, human infection, persistence, research

## Abstract

We report the repeated isolation of *Bordetella petrii* in the sputum of a 79-year-old female patient with diffuse bronchiectasis and persistence of the bacterium for >1 year. The patient was first hospitalized due to dyspnea, which developed into severe cough with purulent sputum that yielded *B. petrii* on culture. After this first episode, the patient was hospitalized an additional 4 times with bronchorrhea symptoms. The isolates collected were analyzed by using biochemical, genotypic, and proteomic tools. Expression of specific proteins was analyzed by using serum samples from the patient. The *B. petrii* isolates were compared with other *B. petrii* isolates collected from humans or the environment and with isolates of *B. pertussis, B. parapertussis, B. bronchiseptica,* and *B. holmesii,* obtained from human respiratory tract infections. Our observations indicate that *B. petrii* can persist in persons with chronic pulmonary obstructive disease as has been previously demonstrated for *B. bronchiseptica*.

## Abstract 2

*B. petrii* infection can persist in persons with chronic obstructive pulmonary disease.

The genus *Bordetella* comprises 9 species; all, except *B. petrii,* are obligatorily associated with host organisms (*1*). The first isolations of *B. ansorpii* were from a cyst (*2*) and from a blood sample (*3*), whereas *B. trematum* has been isolated from infected ears and from wounds in humans. The reservoir and the pathogenic role of these 2 species remain unknown (*4*). *B. pertussis,* a strictly human pathogen, and *B. parapertussis,* a pathogen in both humans and sheep, are agents of whooping cough (*5*). *B. avium* and *B. hinzii* caused respiratory infections in birds and poultry and have also been reported to cause infections in humans (*6**–**9*). The latter 4 *Bordetella* spp. are usually described as extracellular bacteria that secrete adhesins and toxins adapted to their hosts (*10*).

However, 2 other *Bordetella* species, *B. bronchiseptica* and *B. holmesii*, behave differently and are able to persist inside their hosts. *B. bronchiseptica* is a respiratory pathogen which may cause acute or chronic bronchopneumonia and is found in many animals, including dogs, cats, pigs, and rabbits, as well as humans (*11**,**12*). *B. holmesii*, originally described as Centers for Disease Control and Prevenion nonoxidizer group 2 (NO-2), has been isolated from the blood cultures of young adults, mostly with underlying disorders or from sputum (*13**–**15*). The reservoir of this bacterium is unknown. However, *B. petrii* has also been isolated from patients with cystic fibrosis (*16**–**19*). Unlike the “classical” pathogenic species, *B. pertussis* and *B. parapertussis*, *B. petrii, B. holmesii,* and *B. bronchiseptica,* have the ability to acquire or exchange genomic regions (*20**,**21*). *B. petrii* possesses the largest number of huge genomic islands collectively known as integrative and conjugative elements (*22**,**23*). Several determinants of virulence expressed by *B. pertussis*, *B. parapertussis*, and *B. bronchiseptica,* such as filamentous hemagglutinin (FHA), pertactin (PRN), and fimbriae (Fim2 and Fim3), and toxins such as pertussis toxin (PT) and adenylate cyclise-hemolysin (AC-Hly) were not detected in *B. petrii,* except for an FHA-related adhesin with low similarity (*22*). In this study, we describe an immunocompetent adult with predisposing factors (chronic obstructive respiratory disease and local corticotherapy) who acuired an acute *B. petrii* infection that had long-lasting persistence.

## Materials and Methods

### Case History

A 79-year-old nonfebrile woman was hospitalized in October 2007 with dyspnea, which had progressed over 1 week to severe coughing with abundant purulent and hemoptoic sputum, although the patient had been treated with respiratory physiotherapy and corticosteroid aerosols at home (which was in poor hygienic condition). C-reactive protein level was only moderately elevated (27 mg/mL, reference <10 mg/mL). This patient had experienced pulmonary tuberculosis 33 years earlier and had been successfully treated. For 30 years, she had diffuse bronchiectasis, which had required a right middle lobectomy 23 years previously. She had a myocardial infarction 6 years ago. The patient also had severe chronic hyponatremia, diagnosed as Schwartz-Bartter syndrome, which may have been related to a chronic respiratory deficiency. At admission, a purulent sputum sample was taken (class 5 of Bartlett-Murray and Washington criteria) (*24*) yielded a monomicrobial culture (10^8^ CFU/mL) of *B. petrii*. After receiving empirical treatment with amoxicillin-clavulanate, the patient improved slowly and was discharged after 2 weeks. Over the next 12 months, the patient returned with bronchorrhea symptoms in November and December 2007 and in May and November 2008. Each time, *B. petrii* was isolated from purulent sputum specimens in either pure or mixed cultures (in the second sputum culture, *B. petrii* 10^8^ CFU/mL and *Citrobacter freundii* 10^4^ CFU/mL; in the third sputum culture, *B. petrii* 10^8^ CFU/mL and *C. freundii* 10^7^ CFU/mL; in the fourth culture, *B. petrii* 10^7^CFU/mL pure culture; in the fifth culture, *B. petrii* 10^7^ CFU/mL and *Haemophilus parainfluenzae* 10^7^ CFU/mL; and in the last sputum culture, *B. petrii* 10^7^ CFU/mL pure culture). These episodes did not require hospitalization, and the patient’s symptoms were empirically treated with amoxicillin and clavulanate. However, during these repetitive episodes, the patient did not have any general symptoms of sepsis (no fever nor elevated C-reactive protein level). The reactivation of tuberculosis and infection with other mycobacteria were excluded by 3 microscopic examinations and liquid and solid cultures. The patient died in January 2009 from deep electrolytic disorders related to Schwartz-Bartter syndrome.

### Bacterial Isolation, Identification, DNA Extraction, and Growth Conditions

Sputum samples (*25*) were plated onto the following agar plates: chocolate PolyViteX agar for bacterial numeration, bromo-cresol purple (BCP) agar plates, selective *Haemophilus* (chocolate bacitracin) agar, Columbia agar with nalidixic acid and 5% sheep blood (all from bioMérieux, Marcy-l’Etoile, France), and CHROMagar Candida (BBL, Becton, Dickinson and Company, Le Pont de Claix, France). Plates were incubated at 37°C for 24 h and for 48 h in a humidified atmosphere with 9% CO_2_. For each sample, 10^6^–10^8^ CFU/mL were observed and characterized. At 48 h, small colonies of a gram-negative bacterium were detected on the first 3 media. Routine identification was performed by a Gram-Negative Identification card on a VITEK 2 automate (bioMérieux) and manually by using API 20NE, API 32GN (bioMérieux), and RapID NH (Remel, Lenexa, KS, USA) strips in accordance with the manufacturer’s instructions.

Six isolates were collected sequentially from the patient over 13 months. We chose to analyze the first (October 2007), middle (May 2008), and final (November 2008) isolates from the patient and compared them with the other *B. petrii* isolates. The *Bordetella* reference strain and clinical isolates used in this study are listed in Table 1. Bacterial suspensions were prepared from bacteria grown on Bordet-Gengou agar, supplemented with 15% defibrinated blood (BGA, Difco, Detroit, MI, USA) for 24 h at 36°C, which were then resuspended in saline at 1.8 × 10^10^ CFU/mL.

**Table 1:** Reference strains and isolates of *Bordetella* spp. isolates used in this study, October 2007–November 2008

Isolate	Species	Year collected	Origin	Reference
FR3799	*B. petrii*	2007	Human	This study
FR3891	*B. petrii*	2008	Human	This study
FR 3996	*B. petrii*	2008	Human	This study
FR3497	*B. petrii*	1995	Human	(*17*)
KMBW	*B. petrii*	Unknown	Environment	(*1*)
CIP 8132 (Tohama)	*B. pertussis*	1985	Human	(*26*)
Bpp12822	*B. parapertussis*	1993	Human	(*26*)
Bbs RB50	*B. bronchiseptica*	Unknown	Rabbit	(*27*)
Bho1	*B. holmesii*	2007	Human	(*28*)

### Sequencing of the 16S rRNA, *risA,* and *ompA* Genes

DNA from the selected isolates was extracted by using a DNeasy blood and tissue kit (QIAGEN, Courtaboeuf, France). DNA amplification of the 16S rRNA gene was performed as previously described (*29**,**30*). Amplification and sequencing of the genes for the *Bordetella* outer membrane protein A (*ompA*) and the response regulator (*risA*) was performed by using the method described by von Wintzingerode et al. (*1*) with a few modifications. To optimize PCR amplification conditions, we designed primers for the *ompA* gene (ompA3e: 5′-CTC CTC CAA ATT CGC TCT GGC-3′ and ompA4b: 5′-GCA GTT CGC CCT TGC CTT-3′) and *risA* gene (RisA1c: 5′-AAA ACA CCA ATC CCA TCC GC-3′ and RisA2d: 5′-ACA GGT TGA GCA CAT AGG GC-3′).

The nucleotide sequences of 16S rRNA, *risA*, and *ompA* genes from the 3 isolates of the patient (FR3799, FR3891, FR3996) and from the other isolate of human origin (FR3497) have been submitted to the EMBL Nucleotide Sequence Database under the respective accession numbers FN691469/FN691470/FN691471/FN691472; FR669151/FR669152/FR669153/FR669154; and FR669155/FR669156/FR669157/FR669158. For comparative sequence analysis of the 3 different genes, the software, clustalW was used (*31*).

### Matrix-assisted Laser Desorption and Ionization Time-of-Flight (MALDI-TOF) Identification

A MALDI-TOF Axima Assurance (Shimadzu-Biotech Corp., Kyoto, Japan) was used. A positive ion mode, liner mode of detection was running, with a laser frequency of 50 Hz and a mass range window of 2,000–30,000 kDa. The sample was prepared, in duplicate, by a direct deposit of a colony fraction on a target plate, and addition of 1 µL of matrix (acide α-4-cyano-4-hydroxycinnamique, AnagnosTec, Potsdam-Golm, Germany), and drying at ambient temperature. Controls and calibration were done with *Escherichia coli* CCGU 10979 (Culture Collection Göteborg University, Göteborg, Germany). One hundred spectra were obtained, with Launchpad version 2.8 software for spectrum acquisition (Shimadzu-Biotech Corp). Spectra were analyzed with Saramis software, version 3.3.2 (AnagnosTec).

### Antimicrobial Drug Susceptibility Testing

MICs were determined by using Etest strips (AB Biodisk, Solna, Sweden; bioMérieux) in accordance with the manufacturer’s recommendations (EAS 004 2007–6), with 0.5 McFarland inoculum on Mueller-Hinton medium, supplemented with 15% horse blood in an atmosphere of 9% CO_2_ at 37°C (*32**,**33*). *Escherichia coli* ATCC 25922 and *Pseudomonas aeruginosa* ATCC 27853 were used as controls. We tested the following: penicillin, amoxicillin, piperacillin, piperacillin and tazobactam, cefotaxime, ceftazidime, ertapenem, imipenem, meropenem, doripenem, gentamicin, tobramycin, amikacin, levofloxacin, ciprofloxacin, moxifloxacin, minocycline, tygecycline, cotrimoxazole, fosfomycin, quinupristin and dalfopristin, rifampin, daptomycin, linezolid, clindamycin, and fucidic acid.

### Additional Tests

Pulsed-field gel electrophoresis (PFGE) analysis was performed as described by Caro et al. (*34*). Western blot analyses were performed as previously described (*35*). Serum specimens used included polyclonal specific murine serum specimens (anti-FHA, PRN, PT, AC-Hly) (*35*), the serum collected 7 months after the hospitalization of the patient infected with *B. petrii,* and 2 pools of serum samples from patients infected with *B. pertussis* and *B. bronchiseptica,* respectively.

Fimbrial protein expression was detected by agglutination with monoclonal anti-Fim2 and anti–Fim-3 antibodies. Cytotoxicity of bacteria to murine alveolar macrophage J774-*A1* cells was measured as previously described by Bassinet et al. (*36*).

## Results

The bacteria isolated from the patient grew slowly on the chocolate, *Haemophilus* chocolate, and BCP agar plates. On routine media, the bacteria were irregular gram negative coccobacillus, nonmotile, and strictly aerobic. They tested positive for oxidase and catalase, were susceptible to colistin, and grew at 36°C, 25°C, and 42°C.

Automated routine identification showed twice that this bacterium was in the *Moraxella* group (probability 95%). API 32GN identified the bacteria at 24 h as *Methylobacterium mesophilicum* (doubtful identification: 98.4%, with low typicity index [T] = 0.39) and at 48 h as *Acinetobacter,* *Pseudomonas,* or *Achromobacter* (nonreliable identification). API 20NE identified the bacteria (profile code 1001067) with weak discrimination as *Achromobacter denitrificans* (31.1%; T = 0.59) or *B. bronchiseptica* (67.1%; T = 0.66). RapID NH gave *Haemophilus influenzae* as first choice (inadequate identification; probability 76%; bioscore 1/610).

At 48 h to 72 h, cultures on BGA medium showed small, nonhemolytic colonies ≈1 mm in diameter, which were not producing any brown pigment. These tested negative for urease production. The spectra obtained gave a good identification of *B. petrii* (identification agreement 55.4%).

The sequencing of the 16S rRNA confirmed the MALDI-TOF identification. The 16S sequences showed 99% of similarity with the 16S rRNA gene sequences from the type strain of environmental origin and the other isolate from human origin. We then performed the sequencing of *risA* and *ompA* genes to compare with the genes of the other *B. petrii* isolates. The *risA* gene sequences of the clinical isolates showed 93% similarity with the type strain of environmental origin. The species with the next highest similarities were *B. bronchiseptica*, *B. parapapertussis,* and *B. pertussis,* all with a nucleotide identity of 87%. The *ompA* gene sequences of the clinical isolates demonstrated 89% similarity with the type strain of environmental origin. The species with the next highest similarities were *B. bronchiseptica*, *B. parapapertussis*, and *B. pertussis,* all with a nucleotide identity of 86%. The weaker score obtained with *ompA* was due to an insertion of 12 nt versus the environmental strain, nucleotides not present in the other isolates from human origin.

The data regarding antimicrobial drug susceptibility obtained are shown in Table 2. Results are compared with other data available from the literature.

**Table 2:** Antimicrobial drug susceptibility testing for *Bordetella petrii* isolates, by strain and strain type, October 2007–November 2008*

Drug	FR 3799, clinical†	FR3891, clinical†	FR 3996, clinical†	FR 3497, clinical (*17*)	KMRW, environmental (*1*)	Fry et al., clinical (*16*)‡	Stark et al., clinical (*18*)
Penicillin	>32	>32	>32	>32	>32	>32	ND
Amoxicillin	>256	>256	>256	>256	8	>256	+clav: 4
Piperacillin	16	12	12	256	0.25	ND	ND
Piperacillin and tazobactam	16	48	24	256	0.38	2	<4
Cefotaxime	>32	>32	>32	>32	>32	>32	Ceftriax: >64
Ceftazidime	48	256	>256	2	4	32	16
Ertapenem	>32	>32	>32	0.016	0.047	>32	ND
Imipenem	3	4	8	1	0.75	>32	ND
Meropenem	12	>32	12	0.023	0.047	>32	<0.25
Doripenem	32	>32	>32	0.094	0.125	ND	ND
Gentamicin	1	1	1.5	12	3	4	4
Tobramycin	1	1	1	64	4	16	2
Amikacin	8	16	16	64	48	>256	8
Levofloxacin	16	24	24	2	0.25	ND	ND
Ciprofloxacin	>32	>32	>32	4	0.38	>32	2
Moxifloxacin	12	12	12	1.5	0.032	ND	ND
Minocyclin	0.75	0.75	0.75	0.5	0.125	ND	ND
Tygecyclin	0.75	1	1	0.25	0.023	ND	ND
Cotrimoxazole	0.50	0.50	0.50	0.012	0.006	8	ND
Fosfomycin	4	8	8	6	12	ND	ND
Quinupristin and dalfopristin	>32	>32	>32	>32	>32	ND	ND
Rifampin	>32	>32	>32	>32	>32	>32	ND
Daptomycin	>256	>256	>256	>256	>256	ND	ND
Linezolid	>256	>256	>256	>256	>256	ND	ND
Clindamycin	>256	>256	>256	>256	>256	>256	ND
Fucidic acid	>256	>256	>256	>256	>256	ND	ND

*Values are MICs by Etest in μg/mL. +clav, in combination with clavulanic acid; Ceftriax, ceftriaxone tested instead of cefotaxime; ND, not determined. †This study. ‡Ampicillin was tested instead of amoxicillin.

**Figure 1:**
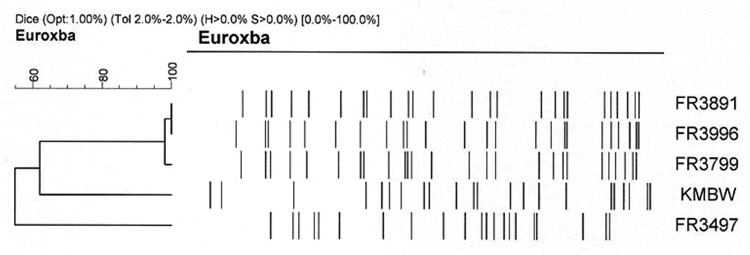
Genomic analysis of *Bordetella petrii* isolates chromosomal DNA profiles obtained after digestion with *Xba*I. Identity of the isolates is indicated.

As shown in Figure 1, the PFGE patterns obtained with the DNA from the 3 isolates of the reported human case-patient are identical, whereas the patterns obtained with the DNA of the other isolate from human origin and the isolate from the environment show several differences. This indicates that the isolates of the present study are related but part of a different PFGE group.

**Figure 2:**
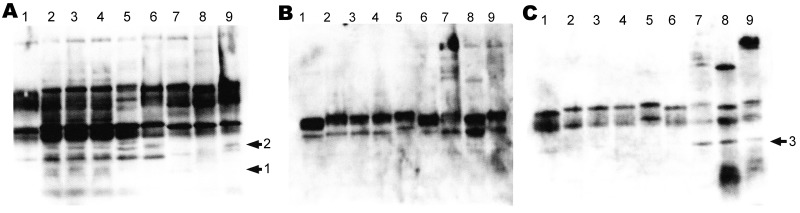
Western blot analysis of 10 μL of bacterial suspension (1.8 × 10^10^ CFU/mL) loaded to a gel and subjected to electrophoresis. The proteins were transferred onto a nitrocellulose membrane, which was incubated in mouse or human serum as described in Materials and Methods. Serum samples used were convalescent-phase serum of the *Bordetella petrii*–infected patient (A), a pool of serum specimens from *B. pertussis*–infected patients (B), and a pool of serum specimens from *B. bronchiseptica–*infected patients (C). Lane 1, *B. holmesii*; lane 2, *B. petrii* FR3799; lane 3, *B. petrii* FR3891; lane 4, *B. petrii* FR3996; lane 5, *B. petrii* FR3497; lane 6, *B. petrii* KMBW; lane 7, *B. pertussis* 8132; lane 8, *B. parapertussis* 12822; lane 9, *B. bronchiseptica* RB50. Arrows indicate the proteins specifically recognized by the anti-serum.

Results were negative for Fim2 and Fim3 by using the agglutination technique as were results for FHA, PRN, PT, and AC-Hly by Western blot (data not shown) with specific antibodies. As shown in Figure 2, the serum of the patient infected with *B. petrii* recognized several proteins in the bacterial suspensions of the 3 *B. petrii* isolates collected from the patient. The same proteins are recognized in the bacterial suspensions of the 2 other *B. petrii* isolates of clinical and environmental origin, except for 1 low-molecular-weight protein. Most of these proteins, with small differences in molecular weights, are also recognized in bacterial suspensions of *B. pertussis*, *B. parapertussis*, *B. bronchiseptica,* and *B. holmesii*, except for 1 protein. The pools of serum samples from patients infected with *B. pertussis* and *B. bronchiseptica* recognized high-molecular-weight proteins expressed by *B. pertussis, B. parapertussis,* and *B. bronchiseptica* in the bacterial suspensions of these 3 species. However, they did not recognize these high-molecular-weight proteins in the bacterial suspensions of *B. petrii* and *B. holmesii*. Finally, the pool of serum samples from patients infected with *B. bronchiseptica* recognized 1 protein in the *B. pertussis, B. parapertussis, B. bronchiseptica* and *B. holmesii* bacterial suspensions but not in the *B. petrii* bacterial suspensions.

In terms of cytoxicity, *B. pertussis* and *B. bronchiseptica* are cytotoxic for the J774-A1 macrophages. However, none of the *B. petrii* isolates were cytotoxic.

## Discussion

Identification of *B. petrii* is still a major problem for clinical laboratories that use automated or manual identification systems. As suggested by Zbinden et al. (*37*) isolates that do not give a 99% or better typing result should be typed by 16S rRNA sequencing or MALDI-TOF. The spectra obtained here with MALDI-TOF gave an acceptable identification of *B. petrii* (identification agreement 55.4%). This is a good score, especially because the database contains only 5 spectra of this recently described species because of the low number of isolates available. The phenotypic characteristics of the isolates in our study are similar to those of the few isolates that have been previously described (*1**,**16**–**19*).

In a previous study on *B. bronchiseptica*, we and others working on *Bordetella* spp (*16*). determined that the results obtained for many antimicrobial drugs using the disk diffusion method correlated poorly with clinical therapeutic results and with MICs established using the reference method ( *32**,**33**;* A. Le Coustumier, unpub. data). Fry et al. (*16*) reported that the clinical isolate was apparently susceptible, by disk diffusion tests, to 5 antimicrobial drugs: clarithromycin, erythromycin, gentamicin, ceftriaxone, and piperacillin+tazobactam. However, the respective reference MICs indicated that only piperacillin+tazobactam was active in vitro with a MIC of 2 µg/mL. Based on the preliminary results the patient received a 6-week course of oral clarithomycin treatment. Despite the successful clinical outcome, the isolate was subsequently shown to be resistant to clarithromycin in vitro. In the only other report (to our knowledge) on a clinical *B. petrii* isolate, MICs were determined by using VITEK2 Compact (bioMérieux) but MICs of drugs for *Bordetella* spp. cannot be determined from this database (*18*). Using Etest strips, a method that has been validated on a wide range of glucose fermenting and nonfermenting gram-negative bacteria, we determined the MIC for 26 widely used antimicrobial drugs from the main therapeutic families (*38*).

All of the 5 isolates in the present study as well as the isolates described by Fry et al. (*16*) and Stark et al. (*18*) appear to have resistance to penicillins (penicillin, amoxicillin), cephalosporins (especially third-generation, extended-spectrum cefotaxime or ceftriaxone and ceftazidime), clindamycin, quinupristin and dalfopristin, rifampin, linezolid, daptomycin, and fucidic acid. We also observed that aminoglycosides had only moderate activity against the bacteria. The isolates in our study also displayed in vitro sensitivity, but low level MICs, to minocyclin, tygecyclin, cotrimoxazole, and fosfomycin.

The large gap in the MICs of amoxicillin and piperacillin between the only environmental isolate available for this study and the clinical isolates may reflect the inducible response to exposure of the clinical isolates to formerly widely used treatment with β-lactams. Tazobactam does not restore the activity of piperacillin, or even degrade it, probably because of the induction of β-lactamase.

In contrast with the isolate of Fry et al. (*16*) and the environmental isolate (*1*), the MICS of carbapenems and systemic fluoroquinolones were high for the isolates from our patient. No previous treatment with carbapenems could be documented from the long medical history of our patient, although fluoroquinolones had been frequently prescribed for bronchiectasis. This lack could be partly due to an impermeability-linked cross-resistance between these 2 chemically unrelated families with the common porin mutation, as frequently has been observed for *Pseudomonas aeruginosa*.

Using PFGE, we observed that the patterns of the DNA restriction fragments for the different isolates collected from the reported human patient were quite similar. This finding confirms the persistence of the same isolate inside the host. However, several differences are observed with the patterns of the DNA from the environmental or human isolates. These differences could be linked to the loss of pathogenic islands in some of the isolates, as has been recently reported (*22*).

Using murine serum samples specific to the major virulence factors expressed by *B. pertussis* and pool of sera from patients infected with either *B. pertussis*, *B. bronchiseptica*, *B. holmesii*, or the serum of the current patient infected with *B. petrii*, we confirmed that *B. petrii* isolates do not express FHA, Fim2 and Fim3, PRN, PT, and AC-Hly. The serum sample from the patient infected with *B. petrii* recognized only 1 protein specific to the *B. petrii* bacterial suspensions derived from the isolates of the clinical patient described in this study. Another protein was specific to the 5 *B. petrii* isolates.

None of the *B. petrii* isolates were cytotoxic for macrophages. This result was likely because these isolates do not express AC-Hly or BteA.

The source of infection and the pathogenic role of *B. petrii* are still unknown. For the study patient, the source of infection, just prior to the first episode, was most likely a contamination that occurred during the aerosol therapy performed at home under poor hygienic conditions (according to the patient). This was potentiated by local corticotherapy.

The prevalence of *Bordetella* spp. within the cystic fibrosis population may well be underestimated, due to the slow growth of this microorganism. However, the prevalence may also be underestimated for all immunosuppressed patients, particularly the elderly. The role that *Bordetellae* spp. such as *bronchiseptica* and *petrii* may play in the progression of pulmonary disease remains unknown, and these species can be misidentified in hospital laboratories (*19*).
